# Ground reaction forces during level ground walking with body weight unloading

**DOI:** 10.1590/bjpt-rbf.2014.0058

**Published:** 2014

**Authors:** Ana M. F. Barela, Paulo B. de Freitas, Melissa L. Celestino, Marcela R. Camargo, José A. Barela

**Affiliations:** 1 Laboratório de Análise do Movimento, Instituto de Ciências da Atividade Física e Esporte, Universidade Cruzeiro do Sul, São Paulo, SP, Brazil; 2 Programa de Pós-graduação em Ciências do Movimento Humano, Instituto de Ciências da Atividade Física e Esporte, Universidade Cruzeiro do Sul, São Paulo, SP, Brazil; 3 Departamento de Educação Física, Universidade Estadual Paulista, Rio Claro, SP, Brazil

**Keywords:** gait, rehabilitation, partial body weight support, kinetics

## Abstract

**Background:**

Partial body weight support (BWS) systems have been broadly used with treadmills as a strategy for gait training of individuals with gait impairments. Considering that we usually walk on level ground and that BWS is achieved by altering the load on the plantar surface of the foot, it would be important to investigate some ground reaction force (GRF) parameters in healthy individuals walking on level ground with BWS to better implement rehabilitation protocols for individuals with gait impairments.

**Objective:**

To describe the effects of body weight unloading on GRF parameters as healthy young adults walked with BWS on level ground.

**Method:**

Eighteen healthy young adults (27±4 years old) walked on a walkway, with two force plates embedded in the middle of it, wearing a harness connected to a BWS system, with 0%, 15%, and 30% BWS. Vertical and horizontal peaks and vertical valley of GRF, weight acceptance and push-off rates, and impulse were calculated and compared across the three experimental conditions.

**Results:**

Overall, participants walked more slowly with the BWS system on level ground compared to their normal walking speed. As body weight unloading increased, the magnitude of the GRF forces decreased. Conversely, weight acceptance rate was similar among conditions.

**Conclusions:**

Different amounts of body weight unloading promote different outputs of GRF parameters, even with the same mean walk speed. The only parameter that was similar among the three experimental conditions was the weight acceptance rate.

## Introduction

Walking is the main way human beings transport their bodies from place to place and it provides functional autonomy. Therefore, acquiring or reestablishing a gait pattern is the main goal for individuals with gait impairments. Among different strategies for walking acquisition or reestablishment, partial body weight support (BWS) systems have been broadly used as a strategy for therapeutic gait training^1^^-^ 6. Most BWS systems consist of a mounting frame and a harness to support a percentage of the individuals' weight as they walk on a motorized treadmill. Only a few studies have investigated the use of this system on level ground walking^5^^,^ 7 ^-^ 13. 

The rationale for using the BWS is that alleviation of body weight might facilitate the walking requirements for individuals with gait impairment and, consequently, promotes a gait pattern close to normal^14^. The treadmill is commonly used because it stimulates rhythmic and repetitive steps^15^ and promotes inter-limb symmetry, both contributing to the improvement of walking temporal characteristics^16^and diminishing the need for propulsive force generation at the end of stance period^17^. However, it has been speculated that the conditions for gait intervention should be as close as possible to daily life activities in order to promote and maximize skills transfer^18^^,^ 19. In this way, one could suggest that the use of the BWS system on ground surface during gait intervention would be more appropriate because it is the condition people encounter on a daily basis. 

Usually, the percentage of BWS on the treadmill ranges from 10% to 70% BWS^1^^,^ 4 ^,^ 14. However, Threlkeld et al.^20^ observed that, in hip, knee, and ankle joint angles, temporospatial gait characteristics of young healthy adults had minimum variation with 10% and 30% BWS and significantly changed with 50% and 70% BWS on a treadmill. Among all these different percentage levels, alleviation of 30% BWS is the most used for individuals with hemiparesis as it yields better results^8^^,^ 15. Although, 30% BWS during level ground walking may hinder the production of force to move the body forward^7^, to our knowledge, no one has systematically investigated the results of ground reaction force (GRF) parameters during level ground walking with different percentages of body weight unloading. 

Patiño et al.^11^ investigated gait characteristics of healthy young adults walking with and without a harness with 0%, 10%, 20%, and 30% BWS on level ground, including the description of the first peak (i.e. weight acceptance), second peak (i.e. push-off), and valley of vertical GRF and the anterior-posterior deceleration and acceleration peaks from one leg. Overall, they found that vertical GRF curves were preserved only when the participants walked without a harness or with harness with 0% BWS, contrary to anterior-posterior GRF curves, which were preserved throughout different experimental conditions. When the participants walked with BWS, they diminished the contact and propulsive forces^11^. Since Patiño et al.^11^ did not control walking velocity throughout the different experimental conditions, it is not possible to conclude how much body unloading could influence these differences, since walking velocity affects GRF components^21^^,^ 22. 

The use of force plates could provide important information concerning accurate and sensitive performance variables that could reveal the effects of walking with BWS on level ground, mainly because BWS is achieved by altering the load on the plantar surface of the foot^23^, and different measurements can be calculated from the GRF components, which reflect differences in kinematic measurements^24^. Consequently, it would be appropriate to describe the effects of body weight unloading during level ground walking in terms of GRF parameters on healthy adults to better implement rehabilitation protocols for individuals with gait impairment with BWS systems. Based on that, in addition to the first and second peaks and valley of vertical GRF and anterior-posterior deceleration and acceleration peaks described previously^11^, it is important to describe additional GRF measurements, such as weight acceptance and push-off rates, impulse, in different conditions and/ or populations^21^^,^ 25 ^-^ 28, keeping walking speed constant. 

The purpose of this study was to describe the effects of body weight unloading on vertical and anterior-posterior GRF parameters in healthy young adults during level ground walking with BWS in order to provide reference values for comparison when planning gait rehabilitation protocols using BWS. It is important to note that the knowledge of the effects of body weight unloading on some kinetic variables would be valuable for those who employ BWS systems as a strategy for gait intervention. 

### Method Sample 

Eighteen healthy young adults (9 males and 9 females) with no apparent gait impairment participated in this study. Their mean (± standard deviation, SD) age, height, and mass were 27±4 years old, 1.66±0.1 m, and 66±14 kg, respectively. This study was conducted in accordance with the Declaration of Helsinki, and it was approved by the Universidade Cruzeiro do Sul Ethics Committee, São Paulo, SP (protocol: CE/UCS-128/2012). All procedures were performed with the adequate understanding and written consent of all participants. None of the participants had previous experience with the BWS apparatus used in the study and all of them wore their own flat shoes during their participation in the study. 

### Instrumentations, task and procedures 

**Figure 1:**
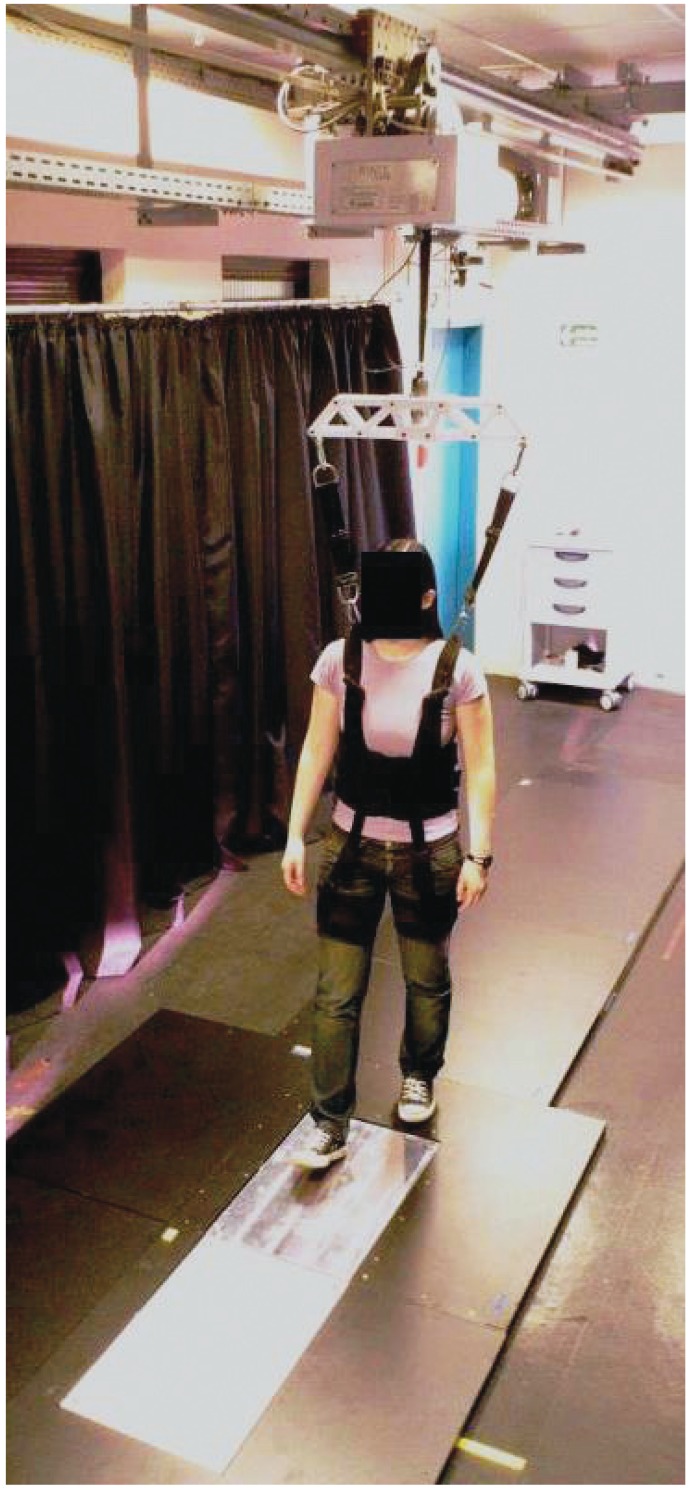
Partial view of the body weight support system employed in the present study and the walkway with built-in force plates. Note: during the experimental session, a thin rubber carpet covered the entire walkway.

The customized BWS system (Finix Tecnologia) used in the present study is shown in Figure 1. It consists of a suspended rail 7 meters long installed 3 meters from the floor and sustained by steel beams, a moving cart, and two electrical servo motors. The moving cart is attached on the bottom of the rail and is moved backward and forward by a belt system linked to a servo motor located at one of the extremities of the suspended rail and controlled by a customized computational routine written in LabView 2011 (National Instruments Inc.), which controls the displacement, velocity, and acceleration of the moving cart. This moving cart has a second servo motor within it, which has a belt and a harness at its other end. Individuals are mechanically supported by the harness, which is pulled up by a belt from the second servo motor. A load cell, positioned between the top of the harness and bottom of the belt, connected to a digital display, provides information about the amount of body weight unloaded. In order to unload the desired amount of body weight, each individual stayed still as one of the experimenters activated the motor to decrease or increase the belt's length. 

Two force plates (Kistler) were embedded into and at the middle of a 7 m long walkway and used for acquisition of ground reaction forces of the left and right lower extremities during the stance periods of a walking cycle. The force plates were connected via charge amplifiers to a laptop and data were acquired via Bioware software (Kistler) at a sampling rate of 240 Hz. 

Before the experimental session, participants were asked to walk freely at a comfortable speed for 15 m approximately as one of the experimenters recorded the time they took to walk the central 10 m, which was used to obtain the mean walking speed. Next, each participant wore the harness and had enough time to become familiar with the task, which consisted of walking with 0%, 15%, and 30% BWS at the speed he/she considered most comfortable. 

The most comfortable speed was recorded by one of the experimenters and it was controlled by the servo motor during the experimental session. 

Prior to the walking performance with the BWS system, each participant stood still on each force plate and their body weight was recorded for calibration purposes. The order of the BWS unloading was randomized, and data from at least three trials for each condition were acquired for further analysis. Trials were considered valid if only one foot had made full contact on each force plate during each step. A digital video camera was used to register which foot landed on each force plate. 

### Data analyses 

Data analyses from both force plates were performed using specific routines written in Matlab (MathWorks, Inc.). These data were digitally filtered using a 4^th ^order, zero-lag Butterworth low-pass filter at 20 Hz and were normalized by the participant's body weight and in time from 0% to 100% of the stance duration. From the vertical GRF component the following variables were calculated: magnitudes of first peak (weight acceptance), second peak (pushoff), and valley (mid-stance); weight acceptance rate (calculated as the magnitude of the first peak divided by the time between initial contact and first peak force); and push-off rate (calculated as the magnitude of the second peak divided by the time elapsed between second peak force and toe-off)^29^. As the peaks are considered the maximum value of the curve before and after the valley, when the vertical component tended to be flat, a visual inspection was made to confirm a correct selection, i.e. the maximum peak during weight acceptance and push-off periods. From the anterior-posterior component, the following variables were calculated: magnitudes of first (deceleration) and second (acceleration) peaks and negative (braking) and positive (propulsive) impulses, calculated as the area under the negative and positive anterior-posterior force component, respectively. Also the mean walking speed that participants walked without the BWS system was compared to the mean walking speed they selected to walk with the BWS system. 

### Statistical analyses 

Data of three repetitions under each experimental condition were averaged for each participant. Statistical analyses involved repeated measures univariate analyses of variance (ANOVA) and multivariate analyses of variance (MANOVA). Except for the first ANOVA that compared the mean walking speed of participants with and without the BWS system, the remaining analyses had as factors leg (right and left) and BWS conditions (0%, 15%, and 30% of BWS). The dependent variables were: weight acceptance, push-off force, and mid-stance vertical GRF valley for the first MANOVA; weight acceptance and push-off rates for the second MANOVA; anterior-posterior deceleration and acceleration peaks for the third MANOVA; and negative and positive impulses for the fourth MANOVA. Post-hoc tests with Bonferroni adjustments were employed to the pairwise comparisons when necessary.An alpha level of 0.05 was used for all statistical tests, which were performed using the Statistical Package for the Social Science software. 

## Results

**Figure 2:**
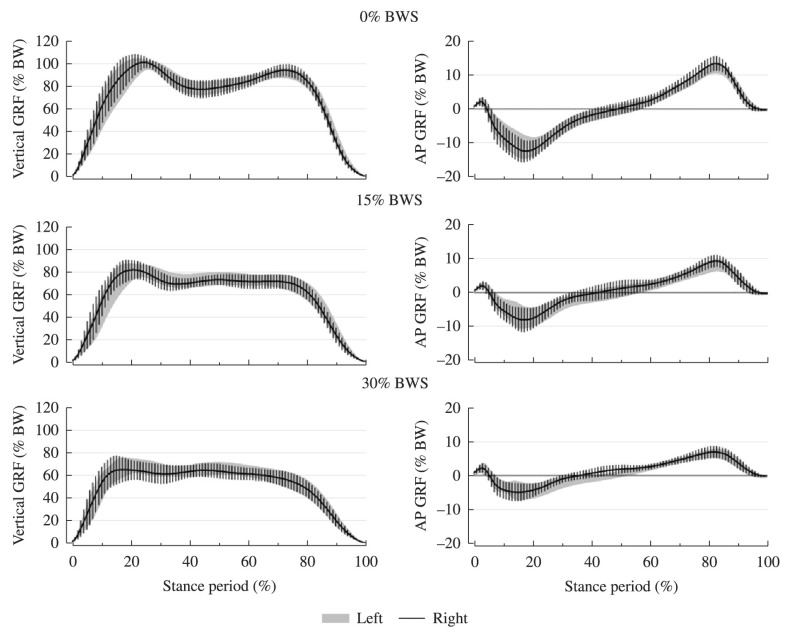
Mean (±SD) time series of vertical and anterior-posterior ground reaction forces (GRF) during stance period for both legs with 0%, 15%, and 30% of body weight support.

All participants walked more slower with the BWS system (1.16±0.12 m/s) compared to their regular walking speed (1.44±0.17 m/s). Figure 2 depicts time series profiles of vertical and anterior-posterior GRF curves during stance period averaged across participants, walking at the three percentages of BWS, and for the right and left leg. A typical vertical GRF pattern of well-defined peaks and valley can be observed when participants walked with 0% BWS. As the percentage of BWS increased, flatter curves emerged, with almost no distinction between the two peaks and valley when they walked with 30% BWS. The typical anterior-posterior GRF pattern, consisting of negative phase followed by positive phase, was observed under the three experimental conditions. 

Table 1 contains the mean (±SD) values of the investigated variables. Peaks and valley from vertical component and deceleration and acceleration peaks from anterior-posterior component decreased as the percentage of BWS increased (*P<*0.001). The only variable that revealed difference between right and left legs was the deceleration peak, in which the left leg presented a higher magnitude than the right leg (*P<*0.005). While no difference was found for weight acceptance rate among the different percentages of BWS (*P>0*.5), the push-off rate decreased as the percentage of BWS increased (*P<*0.001). 

**Table 1:** Mean values (±SD) of first and second peaks and valley of vertical GRF, weight acceptance and push-off rates, and deceleration and acceleration peaks during the stance period of walking with 0%, 15%, and 30% of body weight support (BWS) for right and left legs.

Variables	Leg	0% BWS	15% BWS	30% BWS
Vertical component				
1^st^ peak (% BW)	Right Left	104 (4.65)^a,b^ 104 (5.98)	86 (6.77)^a,c^ 87 (8.06)	73 (7.01)^b,c^ 75 (7.71)
2^nd^ peak (% BW)	Right Left	93 (4.33)^a,b^ 95 (5.08)	76 (4.69)^a,c^ 76 (5.61)	66 (4.88)^b,c^ 67 (5.67)
Valley (% BW)	Right Left	76 (5.87)^a,b^ 75 (6.31)	67 (3.78)^a,c^ 67 (5.57)	60 (4.82)^b,c^ 60 (5.13)
Weight acceptance rate (BW/s)	Right Left	5.78 (1.27) 6.07 (1.57)	5.72 (1.90) 6.05 (1.99)	5.82 (2.05) 5.81 (1.91)
Push-off rate (BW/s)	Right Left	4.66 (0.70)^a,b^ 4.86 (0.87)	3.20 (0.89)^a,c^ 2.89 (0.78)	2.33 (0.52)^b,c^ 2.31 (0.53)
Anterior-posterior component				
Deceleration peak (% BW)*	Right Left	–12.3 (3.33)^a,b^ –12.9 (3.01)	–8.2 (3.08)^a,c^ –9.1 (3.91)	–5.5 (2.14)^b,c^ –6.8 (2.59)
Acceleration peak (% BW)	Right Left	12.7 (1.43)^a,b^ 13.2 (2.07)	8.9 (1.56)^a,c^ 9.3 (1.72)	7.3 (1.44)^b,c^ 7.1 (1.09)

Same letter indicates difference between conditions;

* indicates difference between legs.

**Figure 3:**
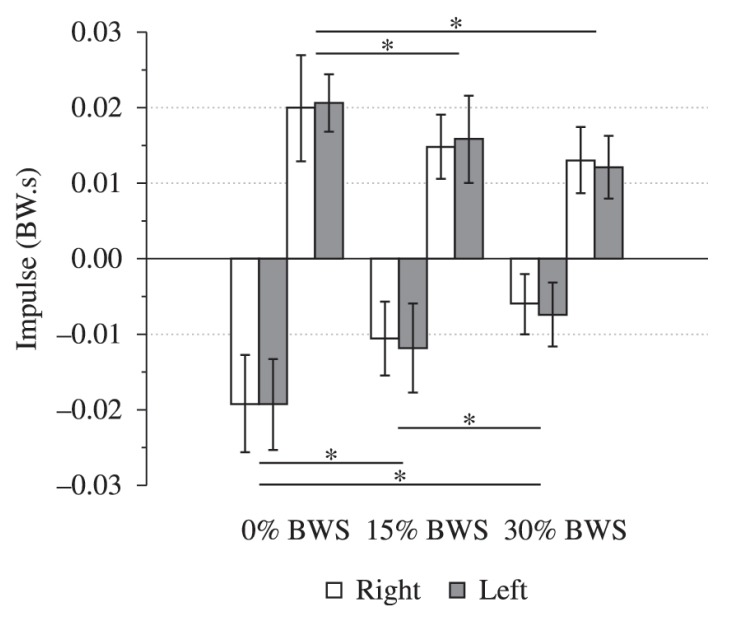
Mean values (±SD) of negative and positive impulses from both legs of all participants walking with 0%, 15%, and 30% of body weight support. * indicates p<0.001.

Figure 3 presents negative and positive impulses for all participants walking under the three percentages of BWS and for the right and left leg. Negative impulse decreased as the percentage of BWS increased (*P<*0.001). Participants generated higher positive impulse when they walked with 0% BWS compared to both 15% (*P<*0.001) and 30% BWS (*P<*0.001) and did not present differences between 15% and 30% BWS (*P>*0.05). 

## Discussion

The purpose of this study was to describe the effects of body weight unloading on vertical and anterior-posterior GRF parameters in healthy young adults during level ground walking with BWS. Overall, the results showed that healthy young adults presented gait alterations due to body weight unloading, although the patterns of vertical and anterior-posterior GRF components were mostly preserved, except for the vertical curve of GRF in the 30% BWS condition that emerged as the flattest curve compared to the 0% and 15% BWS conditions. 

The vertical and the anterior-posterior curves in this study are in accordance with a previous investigation, although Patiño et al.^11^found a flatter curve of the vertical GRF compared to the present study, which might be attributed to a different BWS system and possibly walking speed. In contrast, the mean walking speed remained constant in all experimental conditions for each participant in this study. The increase in body weight unloading explains the flatter shape of the vertical GRF in the 15% and 30% BWS compared to 0% BWS. On the other hand, the shape of the anterior-posterior GRF was maintained among conditions. These results are attributed to the situation to which the participants were exposed, i.e. mechanically supported in the vertical direction, which reduces the gravitational forces acting on both legs and consequently reducing the load that has to be overcome by the performer. In shallow water, for example, walking at a comfortable and self-selected speed, the reduction in speed and apparent body weight influences the shape of both vertical and anterior-posterior GRF curves^30^. 

However, as one walks in shallow water, he/she should deal with the buoyant force that decreases the apparent body weight, and the drag force that increases the resistance to move^30^, differently from the condition with BWS. 

It is known that walking velocity affects the magnitude of GRF peaks^21^^,^ 22. In this way, the gradual reduction in the magnitude of the first and second peaks and the valley as the body weight unloading increased may be attributed specifically to body weight unloading, since the walking velocity was kept constant by the use of the automated BWS system for the three experimental conditions. 

As expected, the magnitude of weight acceptance as well as push-off peaks decreased at approximately the same rate as body unloading (0%, 15%, 30% BWS). In terms of gait rehabilitation, the reduction in weight acceptance may be beneficial because it diminishes the need for generating muscle force that acts on shock absorption and controls limb velocity and body loading at the beginning of the stance period and stabilizes body forward progression. Therefore, individuals who present impaired muscular function due to any neurological or orthopedic disorder could benefit from using this type of system, although this possibility needs further investigation. 

Conversely, the reduction in push-off peak seems to be a drawback of the system given that there is lower muscle force demand for pushing the body upward and forward because the BWS system does it by itself. However, it is important to consider that the propulsive force to move the limb forward during the swing phase must be compensated by the hip muscles^31^. If push-off is usually limited in individuals with gait impairment, the reduction in push-off peak due to BWS may contribute, in the long term, to increased range of motion of hip joints after a period of gait intervention. This aspect was observed previously in individuals with stroke who trained with BWS on level ground^5^. 

Even though the magnitude of weight acceptance and push-off forces decreased as the percentage of body unloading increased, the weight acceptance rate was similar for the three percentages of body unloading and the push-off rate decreased. We could expect that as weight acceptance decreased, weight acceptance rate would decrease as well. However, the magnitude of first peak and the time to reach it decreased as the body unloading increased (Figure 2) due to the action of the BWS system, which was kept at a constant mean velocity. If one takes into account that weight acceptance rate depends on both magnitude of the first peak of the GRF vertical component and time to reach this peak, the weight acceptance rate was similar throughout the three experimental conditions because the rate of first peak magnitude and time to reach this peak was maintained. Similarly, the second peak and the time to reach it decreased as the body weight unloading increased (Figure 2), however, since the rate of the second peak is calculated by dividing the magnitude of second peak by the time elapsed between second peak force and toe-off^29^, the push-off rate decreased as the body unloading increased. Weight acceptance and push-off rates are time dependent^28^, and even though body weight unloading influences the peaks of weight acceptance and push-off from vertical GRF component, only the time of occurrence of the push-off peak was influenced by the manipulation of body weight unloading. 

Regarding the anterior-posterior GRF component, the results revealed that the deceleration and acceleration peaks and the braking and propulsive impulses reduced as the BWS increased. Both the deceleration peak and the braking impulse reduced proportionally more than the body unloading. In the 15% BWS condition, deceleration peak and braking impulse were 69% and 59% (data from right and left leg pulled), respectively, in relation to the 0% BWS condition. In the 30% BWS condition, the deceleration peak and braking impulse were 50% and 36%, respectively, in relation to the 0% BWS condition. The reduction in the deceleration peak and braking impulse could be partially explained by a reduction in both weight acceptance and mean vertical force at the first half of the stance period (data not shown), as the anterior-posterior GRF component (i.e. tangential to the interaction of foot and force plate surface) is directly influenced by the vertical GRF component (i.e. normal to the interaction of foot and force plate surface). There was also a reduction in acceleration peak and propulsive impulse as BWS increased: in the 15% BWs condition, the acceleration peak and propulsive impulse were 69% and 74%, respectively, in relation to the 0% BWS condition; and in the 30% BWS condition, the acceleration peak and propulsive impulse were 58% and 61%, respectively, in relation to the 0% BWS condition. These results could also be partly explained by the reduction in the magnitude of the vertical GRF component. Despite reducing the acceleration peak and propulsive impulse more than the percentage of body unloading, this reduction was lower than the reduction in the braking impulse. 

This study was focused only on GRF data and certainly a more detailed description of level ground walking with BWS including additional analyses (e.g. kinematic and electromyography) should be done. For example, the reduction in the magnitude of the GRF parameters could also be due to different movement strategies (e.g. higher hip flexion) adopted during walking with a BWS system. Unfortunately, our data do not allow us to confirm that. Therefore, in order to understand the effect of body unloading on movement generation, both kinematic and kinetic analyses should be performed simultaneously, and these analyses should be employed in individuals with gait impairment. 

Few studies have assessed individuals with gait impairment as they walked with BWS^7^^,^ 8 ^,^ 13, and to our knowledge, none of them investigated GRF parameters. We did not aim in this study to identify the best conditions for the gait training of individuals with gait impairment. In fact, we aimed to assess the consequences of manipulating body unloading in healthy young adults to provide a normal reference for comparison when preparing gait rehabilitation protocols using BWS. One of the next steps for our group is to investigate vertical GRF parameters during treadmill walking with BWS. 

## Conclusions

Healthy young adults preferred to walk more slowly with BWS on level ground compared to their normal walking speed without BWS. Different amounts of body unloading promote different outputs in terms of GRF parameters, even though the walking speed was maintained among different conditions. The only GRF parameter that was similar among the 0%, 15%, and 30% BWS conditions was the weight acceptance rate. Although it has been established that the BWS system on level ground provides a safe and effective strategy for intervention of patients with stroke^5^, no one to date has investigated the effects of BWS during gait intervention on the GRF parameters of individuals with gait impairment. 
